# Supplementary material for: Targeted Large-Volume Lymphocyte Removal Using Magnetic Nanoparticles in Blood Samples of Patients with Chronic Lymphocytic Leukemia: A Proof-of-Concept Study
Source: Int J Mol Sci. 2023 Apr 19;24(8):7523. doi: 10.3390/ijms24087523 (PMC10139131; doi:10.3390/ijms24087523)

**ONLINE SUPPLEMENT**

**related to**

# Targeted Large-Volume Lymphocyte Removal Using Magnetic Nanoparticles in Blood Samples of Patients with Chronic Lymphocytic Leukemia: A Proof-of-Concept Study

**Stefanie Janker [1,2,†], Simon Doswald [3,†], Roman R. Schimmer [2,4], Urs Schanz [4], Wendelin J. Stark [3], Martin Schläpfer [1,2,‡] and Beatrice Beck Schimmer [1,2,*,‡]**

[1] Institute of Anesthesiology, University Hospital Zurich, University of Zurich, 8091 Zurich, Switzerland; s_janker@hotmail.com (S.J.); martin.schlaepfer@usz.ch (M.S.); beatrice.beckschimmer@uzh.ch (B.B.S.)
[2] Institute of Physiology, University of Zurich, 8057 Zurich, Switzerland; roman.schimmer@usz.ch
[3] Institute for Chemical and Bioengineering, ETH, 8093 Zurich, Switzerland; simon.doswald@protonmail.com (S.D.); wendelin.stark@chem.ethz.ch (W.J.S.)
[4] Department of Medical Oncology and Hematology, University Hospital Zurich, University of Zurich,
8091 Zurich, Switzerland; urs.schanz@bluewin.ch
* Correspondence: beatrice.beckschimmer@uzh.ch
† Equal contribution of S.J. and S.D. as the first authors.
‡ Equal contribution of B.B.S. and M.S. as the last authors.

## ONLINE SUPPLEMENTARY METHODS

### *General experimental details for the production of nanoparticles*

All commercially available reagents were procured from Sigma Aldrich and used as received, when not otherwise stated. The carbon-coated cobalt nanoparticles were purchased from Nanoarmor. The functionalized nanoparticles were analyzed by FT-IR spectroscopy (5% in KBr).

### *CCo@Phenylethanol*

First functionalization of carbon-coated Cobalt (CCo) nanoparticles was adapted from previous described synthesis [1]. In a general procedure, 25 g CCo nanoparticles were dispersed for 5 minutes in 300 ml $H_2O$ in a glass beaker with an ultrasonic bath (Bandelin Sonorex). 3.5 g (25.5 mmol) 4-Aminophenyl alcohol were dissolved in 50 ml deionized water and 10 ml hydrochloric acid. This solution was added to the dispersed particle solution. Seperately, 3.5 g (50.7 mmol) sodium nitrite was dissolved in 30 ml deionized water in an Ice bath and then also added slowly to the particle solution while sonicating. The reaction was carried out for 1 hour in an ultrasonic bath at room temperature. The resulting particles were washed by dispersing them in 300 ml of each 3x $H_2O$, 3x ETOH, 3x Acetone. The washing was done for each cycle as follows: The particles were dispersed in the corresponding solvent for 5 minutes in an ultrasonic bath and subsequently separated with a commercial neodymium magnet. Finally, the obtained CCo@Phenylethanol nanoparticles were dried under *vacuo* at 50°C overnight.

### *CCo@PG*

Hyperbranched polyglycidol synthesis was adapted from previously described procedure [2, 3]. First, the CCo@Phenylethanol is deprotonated and then reacted with glycidol at elevated temperatures. In a typical reaction, 0.6 g CCo@Phenylethanol particles and a magnetic stirring bar were placed in a 2 neck 100 mL round bottom flask and put under protective nitrogen atmosphere. Then, 10 mL of a 2 M Sodium methoxide solution (in Methanol) was added and dispersed in an ultrasonic bath for 20 minutes and then stirred vigorously (1000 rpm) for another 40 minutes. The particles were subsequently washed 4 times with 10 mL dry MeOH

by dispersing the particles in an ultrasonic bath for 5 minutes and magnetic separation. Finally, the particles were dried under high *vacuo*.

The dried and deprotonated CCo@Phenylethanol particles were brought under protective Nitrogen atmosphere. 20 mL dry toluene was added, and the solution dispersed in an ultrasonic bath for 30 minutes. The toluene particles slurry was brought to reaction temperature (90°C) and 10 mL glycidol was added via syringe pump over the course of an hour. The reaction was performed for 16 hours after start of the glycidol addition under constant stirring (600 rpm). Afterwards, the polyglycidol coated CCo particles were magnetically separated from the solution. And the resulted particles were washed with 50 mL of; 1 x toluene,1 x Ethanol, 3 x deionized water, 3x methanol. The washing was done for each cycle as follows: The particles were dispersed in the corresponding solvent for 5 minutes in an ultrasonic bath and subsequently separated with a commercial neodymium magnet. The CCo@PG particles were dried under *vacuo* at 50 °C overnight.

***CCo@PG-COOH***

Succinic anhydride addition was adapted from literature [4]. 0.3 g CCo@PG and a magnetic stirring bar were added in a two neck 100 mL round bottom flask and put under nitrogen atmosphere. 150 mg (1.5 mmol) succinic anhydride was dissolved in 30 mL dry *N,N*-dimethylformamide (DMF) and added to the particle slurry. Finally, 180 mg (1.2 mmol) 4-Dimethylaminopyridine (DMAP) and 1.5 mL (11.9 mmol) trimethylamine in 10 mL DMF was added and the reaction mixture was stirred and heated at 70°C for 16 hours. The particles were washed three times with distilled water (100 mL). The washing was done for each cycle as follows: The particles were dispersed in the corresponding solvent for 5 minutes in an ultrasonic bath and subsequently separated with a commercial neodymium magnet. The CCo@PG-COOH particles were dried under *vacuo* at 50 °C overnight.

***CCo@PG-COO-alemtuzumab conjugate***

Alemtuzumab conjugation was performed via EDC/sulfo-NHC coupling. A 5 mg/mL CCo@PG-COOH particle solution in activation buffer (Ocean Nanotech) was prepared through ultrasonication in an ultrasonic bath. Separately 4 mg/mL 1-Ethyl-3-(3-

dimethylaminopropyl)carbodiimide (EDC) and 2 mg/mL N-hydroxysulfosuccinimide (sulfo-NHS) solutions were prepared in activation buffer. To 200 µL particle solution, 100 µL activation buffer and 10 µL of a 1:1 EDC/sulfo-NHS mixture of the previously prepared solutions were added in an 1.5 mL Eppendorf tube and was homogenized by stepwise vortexing, ultrasonicating and vortexing for each 10 seconds. The solution was afterwards shaken on a thermomixer (Eppendorf) for 10 minutes at 1000 rpm at 25 °C to activate the particles. Alemtuzumab (200 µL, 30 mg/mL) was added and the solution was again homogenized by stepwise vortexing, ultrasonicating and vortexing for each 10 seconds. Then, it was reacted at 1000 rpm for 4 hours at 25 °C in a thermomixer. The reaction was quenched by adding 10 µL quenching buffer (Ocean Nanotech) and homogenized by stepwise vortexing, ultrasonicating and vortexing for each 10 seconds. It was subsequently shaken on a thermomixer (Eppendorf) for 30 minutes at 1000 rpm at 25 °C. The particle-antibody conjugate was magnetically separated with a SuperMag separator (Ocean Nanotech) for 30 minutes at 4 °C and the supernatant removed. The particles were washed three times with 420 µL PBS (4°C, pH 7.4, Life Technologies). Washing cycle was performed by stepwise vortexing, ultrasonicating and vortexing for each 5 seconds. The magnetic separation for each washing cycle was performed in a SuperMag separator (Ocean Nanotech) for 30 minutes at 4°C. The particle-antibody conjugate was finally stored in 420 µL PBS at 4 °C. An analysis is depicted in **Figure S1**, a trans electron microscopy (TEM) picture in **Figure S2**.

***Specificity of nanoparticles***

To determine specificity of nanoparticles, blood of healthy volunteers was subjected to the following treatment options: adding phosphate-buffered saline (PBS), isotype IgG- or anti-CD52-coupled nanoparticles. Experiments were performed as described. After an incubation time the samples were run over a magnetic column, and the remaining lymphocytes were determined by flow cytometry. The tests revealed that IgG-coupled particles did not remove any cells, as demonstrated in **Figure S3**.

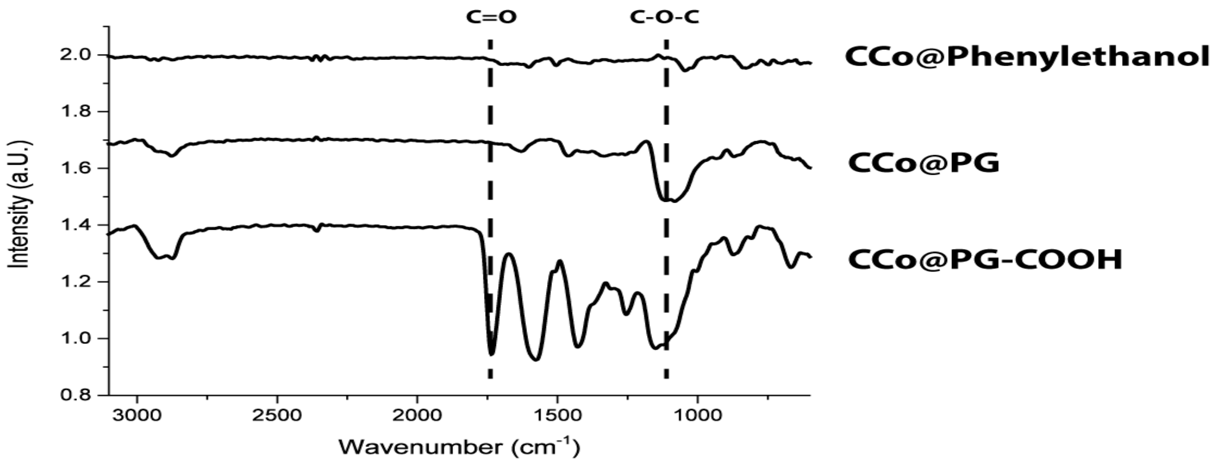


**Figure S1**: Infrared spectra from synthesis steps of magnetic nanoparticle functionalization. The figure is a component of a doctoral thesis of one of the authors [5]. 1125: C-O-C stretch vibration of polyglycidol ether bonds. 1732: Carboxylic acid stretch vibration of succinic acid.

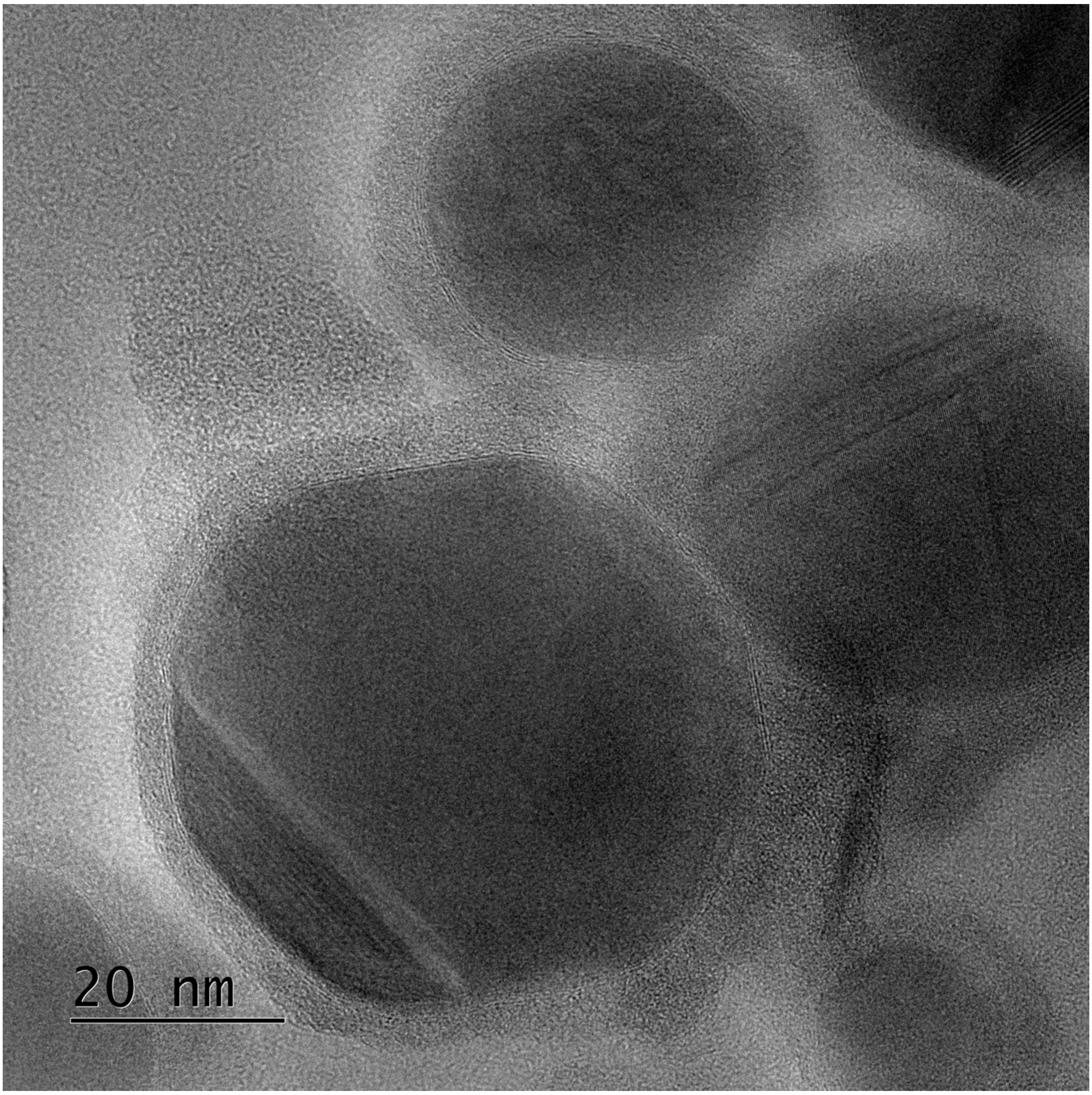


**Figure S2**: Trans electron microscopy (TEM) image of polyglycidol carbon-coated cobalt (CCo) nanoparticles. (Image taken by Dr. Frank Krumeich, Department of Chemistry and Applied Biosciences, ETH Zurich). The image is a component of a doctoral thesis of one of the authors [5].

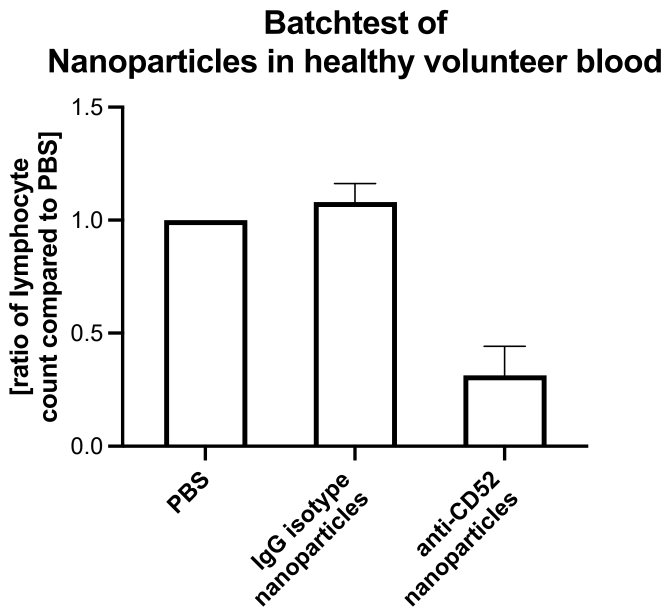


**Figure S3:** Lymphocyte count ratio after exposure to nanoparticles coated with an isotype IgG and with CD52 relative to lymphocytes in the PBS group. n=3 batches tested and used in experiments.

## References


1. Grass RN, Athanassiou EK, Stark WJ: **Covalently functionalized cobalt nanoparticles as a platform for magnetic separations in organic synthesis**. *Angewandte Chemie - International Edition* 2007, **46**:4909-4912.
2. Khan M, Huck WTS: **Hyperbranched Polyglycidol on Si/SiO2 Surfaces via Surface-Initiated Polymerization**. *Macromolecules* 2003, **36**:5088-5093.
3. Zhao L, Chano T, Morikawa S, Saito Y, Shiino A, Shimizu S, Maeda T, Irie T, Aonuma S, Okabe H *et al*: **Hyperbranched Polyglycerol-Grafted Superparamagnetic Iron Oxide Nanoparticles: Synthesis, Characterization, Functionalization, Size Separation, Magnetic Properties, and Biological Applications**. *Adv Funct Mater* 2012, **22**(24):5107-5117.
4. Yang HM, Choi HM, Jang SC, Han MJ, Seo BK, Moon JK, Lee KW: **Succinate Functionalization of Hyperbranched Polyglycerol-Coated Magnetic**

**Nanoparticles as a Draw Solute During Forward Osmosis**. *J Nanosci Nanotechno* 2015, **15**(10):8279-8284.

5. Doswald S: **Functionalization of magnetic nanoparticles for efficient use in biomedical applications**. *Doctoral Thesis.* Zurich, Switzerland: ETH Zurich; 2021.

## TABLE S1: Detailed antibody information for FACS analyses

| | **CD3** | | **CD19** | | **CD52** | |
|---|---|---|---|---|---|---|
| *Catalog #* | MHCD0320 | | MHCD1905 | | MHCD5205 | |
| *Species reactivity* | Human | | Human | | Human | |
| *Host/Isotype* | Mouse / IgG2a | | Mouse / IgG1, kappa | | Mouse / IgG3 | |
| *Class* | Monoclonal | | Monoclonal | | Monoclonal | |
| *Typ* | Antibody | | Antibody | | Antibody | |
| *Immunogen* | Human CD3 epsilon | | Nalm 1 Human cell line | | Human CD52 | |
| *Conjugate* | Alexa fluor® 488 Conjugate | | APC Conjugate | | APC Conjugate | |
| *Concentration* | 0.05 mg/ml | 0.1 mg/ml | 0.05 mg/ml | 0.1 mg/ml | 0.05 mg/ml | 0.1 mg/ml |
| *LOT number* | LOT1987132A | LOT2153684 | LOT2000163A | LOT2054828A | LOT1964662B | LOT2148544 |
| *Producer* | life technologies by Thermo Fisher Scientific, Massachusetts USA | | | | | |

## Table S2 First cohort (n=10) (CD19-positive B lymphocytes)

| | Total lymphocyte concentration (G cells/L) | CD19+ B lymphocytes (G cells/L) | CD19+ B lymphocytes | | |
|---|---|---|---|---|---|
| | | | PBS | 1st cleaning step | 2nd cleaning step |
| 1 | 24.14 | 4.55 | 100% | 46% | - |
| 2 | 68.78 | 28.95 | 100% | 73% | - |
| 3 | 13.79 | 5.32 | 100% | 68% | - |
| 4 | 10.85 | 6.44 | 100% | 34% | 21% |
| 5 | 109.70 | 57.67 | 100% | 93% | 86% |
| 6 | 58.75 | 28.57 | 100% | 88% | 64% |
| 7 | 14.16 | 6.95 | 100% | 31% | 14% |
| 8 | 52.25 | 26.83 | 100% | 79% | 64% |
| 9 | 10.97 | 8.59 | 100% | 41% | 14% |
| 10 | 79.80 | 45.63 | 100% | 72% | 69% |

**Table S2** shows the total number of lymphocytes and the absolute number of CD19+ B lymphocytes in the blood samples of the first cohort. The lymphocyte concentration was determined in the Hematology Laboratory of the University Hospital Zurich, CD19+ B lymphocytes were determined by flow cytometry. The total number of CD19+ B lymphocytes in PBS-treated samples was set to 100% as well as the relative numbers after the first and second cleaning steps.

## Table S3 First cohort (n=10) (CD3-positive T lymphocytes)

| | Total lymphocyte concentration (G cells/L) | CD3+ T lymphocytes (G cells/L) | CD3+ T lymphocytes | | |
|---|---|---|---|---|---|
| | | | PBS | 1st cleaning step | 2nd cleaning step |
| 1 | 24.14 | 0.94 | 100% | 51% | - |
| 2 | 68.78 | 2.56 | 100% | 47% | - |
| 3 | 13.79 | 2.87 | 100% | 44% | - |
| 4 | 10.85 | 1.05 | 100% | 80% | 46% |
| 5 | 109.70 | 5.42 | 100% | 81% | 73% |
| 6 | 58.75 | 0.92 | 100% | 99% | 76% |
| 7 | 14.16 | 1.57 | 100% | 45% | 25% |
| 8 | 52.25 | 0.73 | 100% | 68% | 61% |
| 9 | 10.97 | 1.44 | 100% | 49% | 23% |
| 10 | 79.80 | 1.42 | 100% | 88% | 77% |

**Table S3** shows the total number of lymphocytes and the absolute number of CD3+ T lymphocytes in the blood samples of the first cohort. The lymphocyte concentration was determined in the Hematology Laboratory of the University Hospital Zurich, CD3+ T lymphocytes were determined by flow cytometry. The total number of CD3+ T lymphocytes in PBS-treated samples was set to 100%, as well as the relative numbers after the first and second cleaning steps.

## Table S4 Second cohort (n=11) (CD19-positive B lymphocytes)

| | Total lymphocyte concentration (G cells/L) | CD19+ B lymphocytes (G cells/L) | CD19+ B lymphocytes | | |
|---|---|---|---|---|---|
| | | | PBS | 1st cleaning step | 2nd cleaning step |
| **11** | **63.78** | **29.68** | **100%** | **71%** | **51%** |
| 12 | 19.12 | 9.51 | 100% | 80% | 71% |
| 13 | 9.71 | 3.53 | 100% | 30% | 57% |
| **14** | **97.26** | **41.74** | **100%** | **49%** | **50%** |
| **15** | **93.43** | **40.96** | **100%** | **68%** | **78%** |
| 16 | 6.26 | 1.41 | 100% | 26% | 25% |
| 17 | 12.07 | 4.97 | 100% | 77% | 51% |
| 18 | 12.21 | 5.70 | 100% | 59% | 69% |
| **19** | **77.78** | **26.58** | **100%** | **29%** | **35%** |
| **20** | **42.71** | **24.19** | **100%** | **43%** | **61%** |
| **21** | **51.80** | **23.79** | **100%** | **83%** | **76%** |

**Table S4** shows the total number of lymphocytes and the absolute number of CD19+ B lymphocytes in the blood samples of the second cohort. The lymphocyte concentration was determined in the Hematology Laboratory of the University Hospital Zurich, CD19+ B lymphocytes were determined by flow cytometry. The total number of CD19+ B lymphocytes in PBS-treated samples was set to 100%, as well as the relative numbers after the first and second cleaning steps. Bolt letters indicate patients with >20G lymphocytes/L in whom the nanoparticle concentration was doubled.

## Table S5 Second cohort (n=11) (CD3-positive T lymphocytes)

| | Total lymphocyte concentration (G cells/L) | CD3+ T lymphocytes (G cells/L) | CD3+ T-lymphocytes | | |
|---|---|---|---|---|---|
| | | | PBS | 1st cleaning step | 2nd cleaning step |
| **11** | **63.78** | **1.87** | **100%** | **61%** | **43%** |
| 12 | 19.12 | 0.44 | 100% | 104% | 87% |
| 13 | 9.71 | 0.41 | 100% | 91% | 104% |
| **14** | **97.26** | **3.47** | **100%** | **81%** | **74%** |
| **15** | **93.43** | **1.62** | **100%** | **98%** | **92%** |
| 16 | 6.26 | 0.69 | 100% | 86% | 69% |
| 17 | 12.07 | 0.66 | 100% | 92% | 90% |
| 18 | 12.21 | 0.61 | 100% | 87% | 96% |
| **19** | **77.78** | **1.22** | **100%** | **41%** | **40%** |
| **20** | **42.71** | **0.89** | **100%** | **54%** | **77%** |
| **21** | **51.80** | **1.45** | **100%** | **91%** | **72%** |

**Table S5** shows the total number of lymphocytes and the absolute number of CD3+ T lymphocytes in the blood samples of the second cohort. The lymphocyte concentration was determined in the Hematology Laboratory of the University Hospital Zurich, CD3+ T lymphocytes were determined by flow cytometry. The total number of CD3+ T lymphocytes in PBS-treated samples was set to 100%, as well as the relative numbers after the first and second cleaning step. Bolt letters indicate patients with >20G lymphocytes/L in whom the nanoparticle concentration was doubled.

## Table S6 Second cohort (n=11) (CD52-positive lymphocytes)

| | Total lymphocyte concentration (G cells/L) | CD52+ lymphocytes (G cells/L) | CD52+ positive lymphocytes | | |
|---|---|---|---|---|---|
| | | | PBS | 1st cleaning step | 2nd cleaning step |
| **11** | **63.78** | **9.57** | **100%** | **49%** | **54%** |
| 12 | 19.12 | 5.59 | 100% | 46% | 59% |
| 13 | 9.71 | 4.08 | 100% | 68% | 58% |
| **14** | **97.26** | **21.12** | **100%** | **79%** | **67%** |
| **15** | **93.43** | **52.60** | **100%** | **59%** | **58%** |
| 16 | 6.26 | 2.45 | 100% | 32% | 30% |
| 17 | 12.07 | 4.98 | 100% | 77% | 62% |
| 18 | 12.21 | 6.66 | 100% | 78% | 67% |
| **19** | **77.78** | **18.72** | **100%** | **99%** | **91%** |
| **20** | **42.71** | **17.16** | **100%** | **61%** | **48%** |
| **21** | **51.80** | **28.28** | **100%** | **72%** | **55%** |

**Table S6** shows the total number of lymphocytes and the absolute number of CD52+ lymphocytes in the blood samples of the second cohort. The lymphocyte concentration was determined in the Hematology Laboratory of the University Hospital Zurich, CD 52+ lymphocytes were determined by flow cytometry. The total number of CD52+ T lymphocytes in PBS-treated samples was set to 100%, as well as the relative numbers after the first and second cleaning steps. Bolt letters indicate patients with >20G lymphocytes/Lin whom the nanoparticle concentration was doubled.

*T-Lymphocyte removal in the first and the second cohort*

**First cohort**: While the study focused on B-lymphocytes, the removal efficiency of other CD52 positive lymphocytes was assessed by measuring CD3+ T-lymphocytes. In the first cohort of the study, T-lymphocyte removal was comparable to B-lymphocyte removal. Compared to PBS 65% (p=0.001 vs. PBS) and 54% (p=0.06 vs. the first purification step) of CD3+ T-lymphocytes remained in the blood after the first and the second purification step. Data are illustrated in the supplementary Figure S3.

**Figure S4**

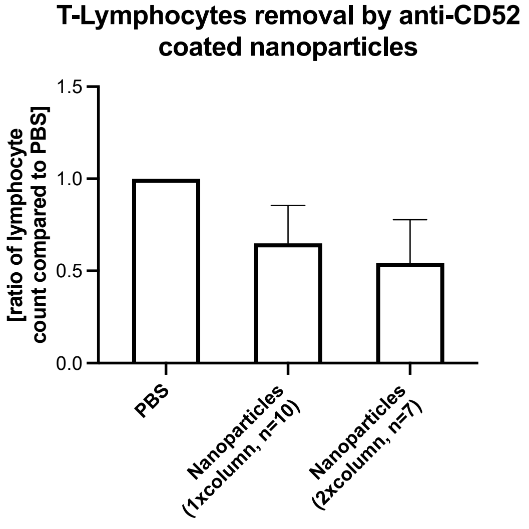


**Second cohort:** Compared to PBS 82% (p=0.01 vs. PBS) and 74% (p=0.22 vs. the first purification step) of CD3+ T-lymphocytes remained in the blood after the first and the second purification step. Data are illustrated in the supplementary Figure S4.

**Figure S5**

**T-Lymphocytes removal by anti-CD52 coated nanoparticles**

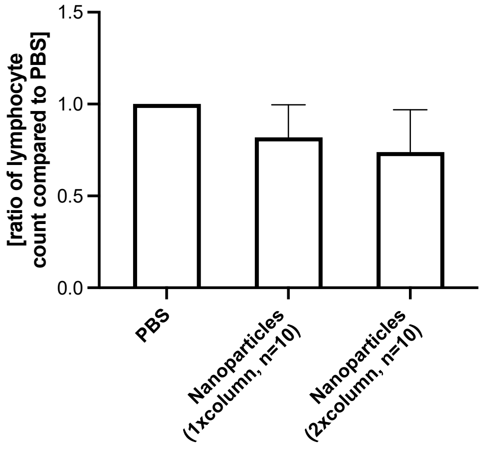

Supplement: Supplementary file 1 [file ijms-24-07523-s001.zip › ijms-2252163-supplementary.pdf]
